# Effect of vegetable consumption with chewing on postprandial glucose metabolism in healthy young men: a randomised controlled study

**DOI:** 10.1038/s41598-024-58103-w

**Published:** 2024-03-30

**Authors:** Kayoko Kamemoto, Yusei Tataka, Ayano Hiratsu, Chihiro Nagayama, Yuka Hamada, Koji Kurata, Michiko Chiyoda, Machi Ito, Masashi Miyashita

**Affiliations:** 1 Institute for Sport Sciences, Waseda University, Saitama, 359-1192 Japan; 2 Graduate School of Sport Sciences, Waseda University, Saitama, 359-1192 Japan; 3 R&D Division, Kewpie Corporation, Tokyo, 182-0002 Japan; 4 Faculty of Sport Sciences, Waseda University, 2-579-15 Mikajima, Tokorozawa, Saitama 359-1192 Japan; 5 School of Sport, Exercise and Health Sciences, Loughborough University, Leicestershire, LE11 3TU UK; 6 Department of Sports Science and Physical Education, The Chinese University of Hong Kong, Shatin, New Territories Hong Kong

**Keywords:** Gastrointestinal hormones, Nutrition

## Abstract

Although thorough chewing lowers postprandial glucose concentrations, research on the effectiveness of chewing vegetables in different forms on postprandial glucose metabolism remains limited. This study examined the effects of vegetables consumed in solid versus puree forms on postprandial glucose metabolism. Nineteen healthy young men completed two 180-min trials on separate days in a random order: the chewing trial involved the consumption of shredded cabbage with chewing and the non-chewing trial involved the consumption of pureed cabbage without chewing. Energy jelly was consumed immediately after the consumption of shredded or puree cabbage. Blood samples were collected at 0, 30, 45, 60, 90, 120 and 180 min. Circulating concentrations of glucose, insulin, total glucagon-like peptide-1 (GLP-1) and glucose-dependent insulinotropic peptide (GIP) concentrations were measured from the plasma. Although plasma glucose concentrations did not differ between the trials, the plasma insulin and GIP incremental area under the curve values were higher in the chewing than in the non-chewing trial. Postprandial total GLP-1 concentrations were higher in the chewing than in the non-chewing trial at 45, 60 and 90 min. This study demonstrates that consuming shredded cabbage while chewing enhances postprandial incretin secretion but has no effect on postprandial glucose concentration.

**Trial registration:** Clinical trial registration ID.: UMIN000052662, registered 31 October 2023.

## Introduction

Chewing is the first process in digestion and involves the crushing of solid food, secretion of saliva and clustering of food particles^1^. Chewing also helps with energy absorption, cephalic phase responses and sensory stimulation^2^. Previous studies have reported that slow and sufficient chewing increases diet-induced thermogenesis^3,4^. Furthermore, poor chewing has been reported to be associated with obesity in adults (for a review, see Tada and Miura. 2018)^5^. Collectively, these findings suggest that thorough chewing influences postprandial metabolism and potentially improves metabolic health.

Several studies have demonstrated that increased chewing before and during meals attenuates postprandial increase in blood glucose concentration in rats^6^ and humans^7–10^. Moreover, thorough chewing was effective in reducing postprandial blood glucose concentrations only in individuals with blood glucose concentrations within the normal range, while no effect of chewing was observed in those with dysglycaemia and a predisposition to type 2 diabetes^7,9^. The incretin hormones, glucagon-like peptide-1 (GLP-1) and glucose-dependent insulinotropic peptides (GIP), are secreted from the small intestine in response to macronutrients ingestion and are responsible for postprandial insulin release^11^. These hormones regulate gastric emptying which helps optimise the process of digestion, resulting in lowered postprandial glucose^12^. Previous studies have reported that gum chewing before a meal enhances the secretion of postprandial GLP-1 and early secretion of insulin^10^. Moreover, increasing the number of chews during meals enhances the early secretion of GLP-1 and GIP^8,13^. Collectively, these studies indicate that increased chewing in healthy adults may stimulate the secretion of incretin hormones and improve postprandial glucose metabolism.

Meal intake sequence typically attenuates the postprandial glycaemic response. In the first place, the effectiveness of “preloading” which involves ingesting nutrients before a meal, has been studied primarily for individuals with type 2 diabetes. Ingestion of whey protein or amino acids before a meal has been shown to stimulate postprandial incretin secretion, enhance insulin secretion, delay gastric emptying and lower postprandial glucose in individuals with type 2 diabetes^14,15^. Another study including individuals with type 2 diabetic and healthy adults reported that eating fish or meat before rice delays gastric emptying, promotes incretin secretion and improves postprandial glucose metabolism^16^. Collectively, consuming protein-rich or fat-rich foods delay gastric emptying and promote incretin secretion. A recent study showed that ingesting vegetables first, followed by meat and rice, attenuates the postprandial glycaemic response by stimulating GLP-1 secretion without stimulating insulin secretion in healthy adults^17^. Although it has been suggested that the high dietary fibre content in vegetables contributes to the improvement of postprandial glucose control, the shape of vegetables when consumed has a different effect on postprandial glucose metabolism^18^. Zhu et al. reported that compared to homogenised vegetables, non-homogenised vegetables delayed the digestion of carbohydrates and mitigated postprandial glycaemic responses more effectively in healthy young women, although this study did not measure incretin hormones, including GLP-1 and GIP^18^. Thus, it remains unclear how chewing influences the two incretin hormones, GLP-1 and GIP, and postprandial glycaemic excursion in healthy individuals .

The purpose of this study was to examine the effects of vegetables consumed in solid versus puree form on postprandial glucose, insulin and incretin concentrations in healthy young men. We selected raw shredded cabbage (with chewing) or cabbage puree (without chewing), particularly raw shredded cabbage, which is a common vegetable in Japanese food culture. We hypothesised that consuming shredded cabbage with chewing would improve postprandial glucose metabolism by stimulating the secretion of incretins and insulin compared with consuming cabbage puree without chewing.

## Methods

### Ethical statement and participants

This study was conducted in accordance with the guidelines of the Declaration of Helsinki. This study was registered with the University Hospital Medical Information Network Center (UMIN), a system for registering clinical trials (31/10/2023, ID: UMIN000052662). Participants of the present study were recruited between July 2021 and November 2021. Following an explanation of the study protocol, food allergy items, and any potential risks that may arise, written informed consent was obtained from 19 healthy young men. The inclusion criteria of the present study were as follows: age > 20 and < 30 years, body mass index < 30 kg/m^2^, not taking any medication or supplementation, no major illness, non-smoking, body mass stable for at least 3 months before the study or no intention to lose weight during the study, no history of immediate allergic reaction to meals (i.e., each food item provided as a test meal in the present study) or no participation in other studies during the conduct of this study. Participants’ physical and descriptive characteristics are presented in Table 1.

**Table 1:** Physical characteristics of the participants.

Characteristics	Mean	(SD)
Age	22	(2)
Height (cm)	173.2	(5.9)
Body mass (kg)	65.5	(9.4)
Body mass index (kg/m^2^)	21.8	(2.5)
Body fat (%)	14.4	(6.0)

Values are mean (SD, standard deviations) for n = 19.

### Standardisation of diet and physical activity

Participants were asked to precisely weigh and record all their food and drink consumed the day before the first trial and to refrain from drinking alcohol during this period. Participants replicated their dietary intake from the first trial in the second trial to ensure that dietary intake was standardised across the trials. Food diaries were analysed by a registered dietitian to determine energy intake and macronutrient content. The participants were asked to remain inactive the day before each main trial and wear a uniaxial accelerometer (Lifecoder-EX; Suzuken Co Ltd., Nagoya, Japan) on the hip to objectively monitor their daily activity during this period. The accelerometer defined 11 levels of activity intensities (0, 0.5 and 1–9), with 0 indicating the lowest intensity and 9 being the highest intensity. A level of 4 corresponds to an intensity of approximately 3 metabolic equivalents (METs)^19^. Levels 1–3, 4–6 and 7–9 correspond to light, moderate and vigorous physical activity, respectively. Activity levels were calculated as light-, moderate-, vigorous-, moderate to vigorous-intensity physical activity and total physical activity. In addition, total (i.e., gross) energy expenditure and total step count (steps per day) were obtained from a Lifecorder using computerised software (Lifelyzer 05 Coach, Suzuken Co Ltd., Nagoya, Japan).

### Anthropometry

Body mass and body fat percentage were measured to the nearest 0.1 kg and 0.1% respectively using a digital scale (TANITA MC780, Tanita Corporation, Tokyo, Japan). Height to the nearest 0.1 cm was measured using a stadiometre (YS-OA, Yoshida Seisakusho Ltd., Gifu, Japan). Body mass index was calculated by dividing body mass in kilogrammes by the square of height in metres.

### Experimental protocol

**Figure 1:**
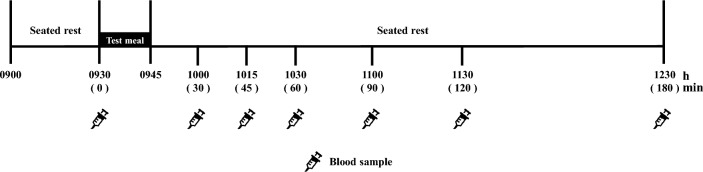
A schematic illustration of the study protocol.

This study used a crossover design. The participants completed two laboratory-based trials in a random order: (1) chewing and (2) non-chewing. The interval between trials was at least six days. The primary outcome of this study was postprandial glucose concentrations, while the secondary outcomes were postprandial insulin, total GLP-1 and GIP. The lead investigator generated the randomisation sequence using a computer-generated random number. A schematic representation of the study protocol is shown in Fig. 1.

After at least 10 h of overnight fasting, each participant reported to the laboratory at 0900 and their body mass was measured. Following a 30-min seated rest, an indwelling catheter was inserted into the vein of the nondominant arm and a baseline blood sample was drawn. The participants then consumed a standardised test meal, with details further discussed in the succeeding section. Shredded cabbage (SALAD CLUB, INC., Tokyo, Japan), mineral water (Suntory natural mineral water, Suntory Holdings Limited, Osaka, Japan), pure lemon juice (POKKA lemon 100, POKKA SAPPORO Food & Beverage Ltd., Aichi, Japan) and energy jelly (In Jelly, Morinaga & Co., Ltd., Tokyo, Japan) were used as the test meal. For the chewing trial, six equal portions of shredded cabbage (1.8 g/kg body mass) were divided into six plates. Participants were instructed to eat the prepared shredded cabbage on each plate in two min at a rate of 88 chews/min (but not the amount of mouthful) using a metronome (SPM300, Seiko Watch Corporation, Tokyo, Japan), completing the meal in 12 min. Additionally, while eating the cabbage, participants were asked to drink 1.2 g/kg body mass of lemon water. In the non-chewing trial, the puree was consumed in 12 min without chewing. The puree was prepared using a blender (VITA-PREP3, Vita-Mix Corporation, Ohio, USA), and the ingredients were shredded cabbage, mineral water and pure lemon juice. The amount of ingredients was adjusted to be the same as that in the chewing trial, and the mixing time was consistent (i.e., 120 g of shredded cabbage was blended for 80 s). Immediately after the consumption of the test meal in both trials, 5.5 g/kg body mass of energy jelly was consumed in 3 min; therefore, the total meal duration was 15 min. The macronutrient composition per 100 g of energy jelly contained 0 g protein, 0 g fat, 25 g carbohydrate and 418 kJ of energy. All test meals were stored in a refrigerator at 4 °C and removed from the refrigerator just before serving. None of the participants reported nausea or gastrointestinal discomfort during or after the test meal. Blood samples were obtained at 30, 45, 60, 90, 120 and 180 min after the first bite of the test meal while the participants were asked to remain in a resting state in the laboratory. During this period, 100 mL of water was provided to the participants every 60 min from the baseline measurements.

### Blood collection and analysis

Venous blood samples were collected in tubes containing clotting activators for serum isolation (Venoject 2, Terumo Corporation, Tokyo, Japan). Thereafter, samples were allowed to clot for 30 min at room temperature and then centrifuged at 1861 × *g* at 4 °C for 10 min to isolate the serum. Serum was then removed into aliquots and stored at − 80 °C until analysis for total cholesterol (Total-C), low-density lipoprotein cholesterol (LDL-C), high-density lipoprotein cholesterol (HDL-C), triglycerides (TG), non-esterified fatty acids (NEFA), total protein (TP) and creatine kinase (CK). The serum concentrations of Total-C, LDL-C, HDL-C and TG were measured by enzymatic colorimetric assays method (Total-C: Total-C Determiner L TC II, Hitachi Chemical Diagnostics Systems Co. Ltd., Tokyo, Japan; LDL-C: Matabo Lead LDL-C, Hitachi Chemical Diagnostics Systems Co. Ltd., Tokyo, Japan; HDL-C: Matabo Lead HDL-C, Hitachi Chemical Diagnostics Systems Co. Ltd., Tokyo, Japan; TG: Pure Auto S TG-N, Sekisui Medical Co. Ltd., Tokyo, Japan; NEFA: NEFA-HR, FUJIFILM Wako Pure Chemical Corporation, Osaka, Japan). Serum TP concentration was determined in Biuret method (TP-HRII, FUJIFILM Wako Pure Chemical Corporation, Osaka, Japan) and CK was measured by the Japan Society of Clinical Chemistry transferable method (FUJIFILM Wako Pure Chemical Co., Osaka, Japan). The intra-assay coefficients of variation were 0.7% for Total-C, 0.9% for LDL-C, 1.0% for HDL-C, 1.3% for TG, 1.1% for NEFA, 1.1% for TP and 1.2% for CK. Venous blood samples were collected in tubes containing sodium fluoride-EDTA (Venoject 2, Terumo Corporation, Tokyo, Japan) to measure plasma glucose and dipotassium salt-EDTA (Venoject 2, Terumo Corporation, Tokyo, Japan) to measure plasma insulin, GIP and total GLP-1. The tubes were immediately centrifuged at 1861 × *g* for 10 min at 4 °C to isolate plasma. Subsequently, the plasma was separated, aliquoted and stored at − 80 °C until the assays were performed. Enzymatic colourimetric assays were performed to measure the plasma glucose concentrations (GLU-HK(M), Shino-Test Corporation, Kanagawa, Japan). Plasma insulin, GIP and total GLP-1 concentrations were measured by enzyme-linked immunosorbent assay (ELISAs) method (insulin: Mercodia Insulin ELISA, Mercodia AB, Uppsala, Sweden; GIP: EZHGIP-54 K, EMD Millipore Corporation, Massachusetts, USA; total GLP-1: YK161, Yanaihara Institute Inc., Shizuoka, Japan). The intraassay coefficients of variation were as follows: 0.8%; insulin, 2.6%; GIP, 5.0% GIP and 4.1%, total GLP-1.

### Sample size calculation

We calculated the required sample size based on data from a previous study^7^ using G*Power 3.1.9.6^20^. The previous study reported that postprandial glucose concentrations measured at 90 min after consuming a standardised test meal were significantly lower in the 30 s chewing trial than the 10 s chewing trial (5.8 ± 0.3 vs 6.5 ± 0.4 mmol/L, *P* < 0.05, mean ± standard error) in adults with the normal glucose tolerance^7^. Analysis of the previously mentioned data using a paired t-test indicated that an estimated total sample size of 18 was needed to provide 80% power to detect significant differences in chewing on postprandial plasma glucose in healthy participants, with an alpha set at 0.05 and a correlation of 0.5. This sample size estimation was powered to detect an effect size of 0.5 (Cohen’s d), using a paired *t*-test for comparison between the two trials. Based on these calculations, 19 participants were recruited for this study.

### Statistical analysis

All data analyses were performed using the SPSS Statistics version 26 statistical analysis software (IBM Corporation, New York, USA). The incremental area under the curve (iAUC) was calculated using GraphPad Prism version 9.2.0 for Windows (GraphPad Software, California, USA). Normality was assessed using the Kolmogorov–Smirnov test. Physical activity data were compared between trials using the paired Student’s *t*-test or the nonparametric Wilcoxon test. Generalised estimating equations were used to examine the between-trial differences in glucose, insulin, GIP and total GLP-1 over time.Where a significant trial-by-time interaction was found, post-hoc pairwise comparisons were performed using the Bonferroni method. Where statistically significant differences in baseline values were found, statistical analysis was performed by adjusting for baseline covariates. Generalised estimating equations were used to compare the baseline values for all blood parameters and the iAUC for glucose, insulin, GIP and total GLP-1 between trials. The 95% confidence intervals (95% CI) for the mean absolute pairwise differences between trials were calculated using the *t*-distribution and degrees of freedom (*n*–1). Effect sizes (ES) (Cohen’s d) were calculated to describe the magnitude of the differences between trials. Effect sizes of 0.2 are considered the minimum important difference in all outcome measures, 0.5 moderate and 0.8 large^21^. Statistical significance was set at *P* < 0.05. All results are presented as mean ± standard deviation (SD). Except for plasma insulin concentration (*n* = 18), no missing values were present for any of the outcome parameters.

### Ethical approval

The study was reviewed and approved by the Ethics Committee on Human Research of Waseda University (approval number: 2021-103).

## Results

### Free-living energy intake and physical activity

All participants reported that they consumed identical foods and drinks on the day before the two trials. The mean self-reported energy intake on the day prior to each trial was 8.9 ± 2.1 MJ/day. Energy intake equated to 13.4% (71.9 ± 25.1 g/day) from protein, 31.3% (74.3 ± 26.7 g/day) from fat and 55.3% (296.4 ± 91.4 g/day) from carbohydrate. The total step counts recorded the day before the trials did not differ between the chewing (9163 ± 6236 steps/day) and non-chewing (10,622 ± 5242 steps/day) trials (95% CI − 4513 to 1596 steps/day, *P* = 0.329). Accelerometer recorded frequencies for moderate to vigorous-intensity physical activity (chewing, 35.5 ± 30.6 min/day; non-chewing, 38.8 ± 26.6 min/day; 95% CI − 15.8 to 9.1 min/day, *P* = 0.578) and total energy expenditure (i.e., gross) (chewing, 9.1 MJ/day; non-chewing, 9.5 MJ/day, *P* = 0.469, median) did not differ between the chewing and non-chewing trials.

### Baseline blood parameters

The baseline blood parameters are shown in Table 2. Plasma GIP and serum CK concentrations were lower in the non-chewing than the chewing trial (73.1 ± 40.9 vs. 96.6 ± 57.2 pg/mL, 95% CI − 42.7 to − 4.3 pg/mL, *P* = 0.010, ES = 0.47; 146.5 ± 66.3 vs. 173.9 ± 96.9 U/L, 95% CI − 55.5 to 0.6 U/L, *P* = 0.036, ES = 0.33, respectively). No other parameters showed significant differences at baseline between the trials.

**Table 2:** Baseline values of blood parameters in the chewing and non-chewing trials.

	Chewing		Non-chewing		95% confidence intervals			*P* value
	Mean	(SD)	Mean	(SD)				
Glucose (mmol/L)	4.8	(0.3)	4.8	(0.2)	− 0.1	–	0.1	0.843
Insulin (pmol/L)	20.4	(12.8)	21.2	(10.6)	− 3.8	–	2.4	0.644
Total GLP-1 (pmol/L)	3.0	(2.8)	3.1	(4.3)	− 0.9	–	0.7	0.792
GIP (pg/mL)	96.6	(57.2)	73.1	(40.9)	4.2	–	42.7	0.010
Total-C (mmol/L)	4.4	(0.7)	4.4	(0.7)	− 0.2	–	0.1	0.378
LDL-C (mmol/L)	2.5	(0.7)	2.5	(0.7)	− 0.2	–	0.1	0.346
HDL-C (mmol/L)	1.5	(0.2)	1.5	(0.3)	− 0.1	–	0.1	0.689
TG (mmol/L)	0.8	(0.5)	0.8	(0.3)	− 0.2	–	0.2	0.771
NEFA (mmol/L)	0.48	(0.15)	0.43	(0.17)	− 0.03	–	0.12	0.235
TP (g/dL)	7.2	(0.4)	7.2	(0.5)	− 0.1	–	0.1	0.647
CK (U/L)	173.9	(96.9)	146.5	(66.3)	− 0.6	–	55.5	0.036

Values are mean (SD, standard deviations) for *n* = 18 (insulin) and *n* = 19 (all others). Means were compared using generalised estimating equations.

*Total GLP-1* total glucagon-like peptide-1, *GIP* glucose-dependent insulinotropic peptide, *Total-C* total cholesterol, *LDL-C* low-density lipoprotein cholesterol, *HDL-C* high-density lipoprotein cholesterol, *TG* triglycerides, *NEFA* non-esterified fatty acids, *TP* total protein, *CK* creatine kinase.

### Blood parameter response

**Figure 2:**
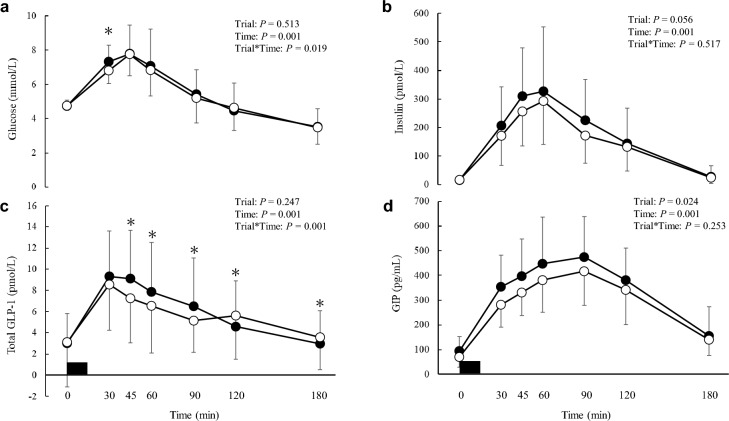
Postprandial plasma glucose ((**a**), *n* 19), insulin ((**b**), *n* 18), total GLP-1 ((**c**), *n* 19) and GIP ((**d**), *n* 19) concentrations across all time points after the first bite of the meal (—●—, the chewing trial; —○—, the non-chewing trial). The black rectangle indicates the time that the test meal was consumed. Values are means ± standard deviation. Values are compared using generalised estimating equations. Post-hoc analysis was adjusted for multiple comparisons using the Bonferroni method. *Significantly different between trials, *P* = 0.026 (for glucose (**a**)) and *P* ≤ 0.049 (for total GLP-1 (**c**)). 0, pre-meal; 30, 30 min after the first bite of the test meal; 45, 45 min after the first bite of the test meal; 60, 60 min after the first bite of the test meal; 90, 90 min after the first bite of the test meal; 120, 120 min after the first bite of the test meal; 180, 180 min after the first bite of the test meal; *total GLP-1* total glucagon-like peptide-1, *GIP* glucose-dependent insulinotropic peptide.

**Figure 3:**
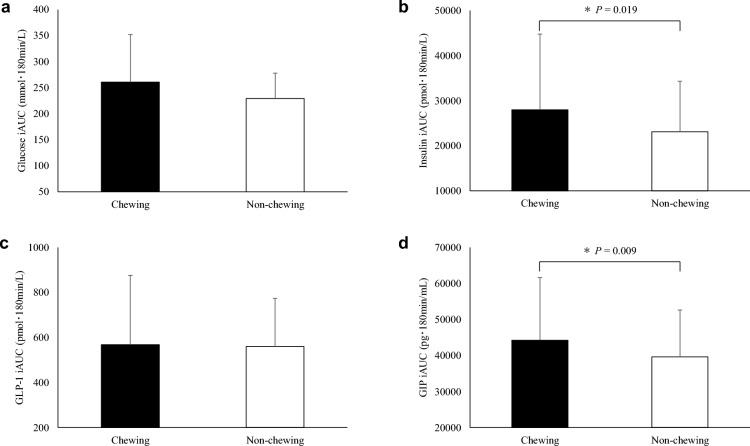
The incremental area under the curve (iAUC) values during the observation period for plasma glucose ((**a**), *n* 19), insulin ((**b**), *n* 18), total GLP-1 ((**c**), *n* 19) and GIP ((**d**), *n* 19) concentrations. Values are means ± standard deviation. Values are compared using generalised estimating equations. *Significantly different between trials. *total GLP-1* glucagon-like peptide-1, *GIP* glucose-dependent insulinotropic peptide.

Plasma glucose, insulin, total GLP-1 and GIP concentrations and their corresponding iAUC values during the observation period are shown in Figs. 2 and 3, respectively. There was a main effect of time (*P* = 0.001) and trial-by-time interaction (*P* = 0.019) on plasma glucose concentration (Fig. 2a). Post-hoc analysis revealed that plasma glucose concentrations were higher in the chewing than in the non-chewing trial at 30 min after the first bite of the test meal (95% CI 0.1 to 0.8 mmol/L,* P* = 0.007, ES = 0.62). There was a main effect of time on plasma insulin concentration (*P* = 0.001). There was no trial-by-time interaction (*P* = 0.517) or main effect of the trial (*P* = 0.056) on plasma insulin concentration (Fig. 2b). The iAUC values during the observation period for plasma insulin were higher in the chewing than in the non-chewing trial (95% CI 275.4 to 9417.1 pmol·180 min/L, *P* = 0.019, ES = 0.34) (Fig. 3b). There was a main effect of time (*P* = 0.001) and trial-by-time interaction (*P* = 0.001) on plasma total GLP-1 concentration (Fig. 2c). Post-hoc analysis revealed that plasma total GLP-1 concentrations were higher in the chewing than in the non-chewing trial at 45, 60 and 90 min after the first bite of the test meal (95% CI 0.7 to 3.1 pmol/L, *P* = 0.002, ES = 0.43; 95% CI 0.2 to 2.4 pmol/L, *P* = 0.017, ES = 0.28; 95% CI 0.0 to 2.7 pmol/L, *P* = 0.049, ES = 0.36, respectively). Further post-hoc analysis revealed that plasma total GLP-1 concentration was lower in the chewing than in the non-chewing trial at 120 and 180 min after the first bite of the test meal (95% CI − 2.0 to − 0.1 pmol/L, *P* = 0.027, ES = 0.1; 95% CI − 1.1 to − 0.1 pmol/L, *P* = 0.023, ES = 0.19, respectively). There was a main effect of trial (*P* = 0.024) and time (*P* = 0.001) on plasma GIP concentration. There was no trial-by-time interaction (*P* = 0.253) for plasma GIP concentration (Fig. 2d). The iAUC values during the observation period for GIP were higher in the chewing than in the non-chewing trial (95% CI 831.8 to 8380.2 pg·180 min/mL, *P* = 0.009, ES = 0.30) (Fig. 3d).

## Discussion

This study demonstrates that vegetables consumed in the puree form without chewing and in the solid form with chewing have no effect on the suppression of postprandial glucose elevation; however, chewing enhanced postprandial insulin and incretin secretion in healthy young men.

Previous studies have shown that increasing the number of chews or prolonged chewing during meals attenuates postprandial glucose concentrations^7,9^. However, consuming a solid form of cabbage with chewing or a puree form of cabbage without chewing did not affect postprandial glucose concentrations in the present study. Madhu et al. (2019) reported that thorough chewing (i.e., chewing 40 times per mouthful) attenuated postprandial glucose concentration compared with chewing at a normal routine frequency in individuals with normoglycaemia^9^. Although the speed of ingestion and the chewing rhythm of the test meal were controlled, the amount of mouthful was not controlled in the chewing trial in the present study resulting the total number of chews per plate varied between participants, which may have influenced the postprandial glucose concentration. However, studies have reported no effect on changes in postprandial glucose concentration despite specifying the number of chewing times per mouthful and increasing the number of chewing times^13,22^. The participants in these studies were healthy young adults, similar to those in the present study. Thus, it is also possible that compared with vegetables consumed in the puree form without chewing, vegetables consumed in solid form with chewing may not have resulted in a reduction in postprandial blood glucose concentration in young healthy individuals. Furthermore, differences in the test meals and study design may have caused inconsistencies in the results. The importance of chewing is influenced by the hardness and dryness of food; hard and dry foods enhance saliva secretion and promote bolus formation with adequate chewing^1^. A previous study using groundnuts as the test meal reported that thorough chewing attenuated postprandial glucose concentrations^9^. Thus, the form of food before swallowing may influence metabolic responses. In the present study, the effect of chewing on postprandial glucose concentrations may have been diminished, as only shredded cabbage was chewed before consuming jelly food that required minimal or no chewing.

The findings of enhanced incretin secretion with the consumption of vegetables in the chewing trial compared to the non-chewing trial in the present study are consistent with previous studies reporting that increasing the number of chews during meals enhances the early secretion of incretin hormones (i.e., GIP and total GLP-1) in healthy individuals^8,13^. Interestingly, the present study showed that increased GIP and insulin concentrations during the chewing trial were observed throughout the 180-min postprandial period. Zhu et al. (2013) has reported that increasing the number of chews in the meal enhanced early (15-min postprandially) increase in GIP and insulin in healthy men^8^. Another study demonstrated that increasing the number of chews increased GLP-1 concentrations, 30–120 min after a meal in healthy lean individuals^13^. The present study extends these previous findings^8,13^ by demonstrating that chewing vegetables before energy jelly augments GIP and insulin concentrations over the postprandial period. GIP and GLP-1 are gastrointestinal hormones that are secreted in response to nutrient intake and promote insulin secretion; GIP has also been identified in the pancreas^23^. Postprandial release of GLP-1 maintains glucose homeostasis and overall energy status^24^. GLP-1 inhibits hepatic gluconeogenesis in the liver through activation of GLP-1 receptor on the vagal afferents innervating the hepatic portal vein^25^. Inhibition of gluconeogenesis in the liver is independent of GLP-1 function in the pancreatic islets^26^, and GLP-1 has attracted attention for its role in improving glucose tolerance. Furthermore, GLP-1 has anorexigenic effects and reduces food intake^27^ although this was not examined in the present study. A previous study reported that gum chewing enhanced GLP-1 secretion and increased satiety in a fasted state^28^, whereas another study reported that an increase in the number of chews enhanced postprandial GLP-1 secretion but not subjective appetite^13^. Thus, the relationship between chewing-induced enhanced GLP-1 secretion and subjective appetite remains unclear. It would be interesting to examine whether acute vegetable consumption with chewing augmented postprandial incretin secretion, as observed in the present study, contributes to the regulation of glucose and energy homeostasis after consuming multiple meals throughout the day.

Consuming vegetables containing dietary fibre at the beginning of a meal attenuated the increase in postprandial glucose concentrations^17^. A diet high in dietary fibre is not only effective in preventing lifestyle-related diseases such as obesity and type 2 diabetes^29^, but it has also been reported that better glycaemic control was achieved with long-term intervention of the meal sequencing method of consuming vegetables before carbohydrates in individuals with type 2 diabetes^30^. While dietary fibre increases food transit time and delays gastric emptying^31^, preloading fat or protein together with dietary fiber attenuates postprandial glucose concentrations^32^. For instance, recent findings on meal sequencing for glycaemic control have examined the timing of protein and fat intake to stimulate GLP-1 secretion, and have shown that protein and fat intake with dietary fibre before carbohydrate intake is a more effective meal sequence approach to attenuate the elevation of postprandial blood glucose concentration^32^. The present study demonstrates that the consumption of fibre-rich vegetables with chewing prior to carbohydrate ingestion (i.e., energy jelly) enhanced early total GLP-1 secretion compared to consumption without chewing. However, in the present study, no significant effect of vegetable consumption with chewing was observed on the elevation of postprandial blood glucose concentration. This could be attributed to the fact that an energy jelly containing only carbohydrates instead of a standard diet (i.e., a variety of nutrients, including protein and fat) was used as the test meal, which may not have reached to the apparent suppression of the postprandial blood glucose concentration. Thus, further research is required to clarify the effectiveness of chewing with respect to glycaemic control.

This study had several strengths. Previous studies focusing on chewing^7–9,13,22^ and meal sequences^17,32^ have been conducted independently as method of controlling postprandial glucose metabolism. The present study focused on two spontaneous factors: chewing and the sequence of food intake, with and without chewing vegetables before energy jelly. The findings from our study suggest that chewing and ingesting vegetables before energy jelly stimulates the additional secretion of incretin and insulin. The present study had several limitations. First, we recruited a small group of healthy young men, which may have limited the application to other population groups. In a recent study, healthy young women had significantly higher GLP-1 responses to duodenal glucose infusion than men, suggesting the further empirical research on women^33^. Second, we did not examine the habitual number of chews per participant. Postprandial glucose concentration is significantly decreased in thorough chewing trials compared to routine chewing trials in normoglycaemic individual^7,9^. Since the participants in this study were healthy men with normoglycaemia and the habitual number of chews was undetermined, it was not possible to determine whether sufficient chewing had occurred before swallowing during the chewing trials. Finally, the present study employed energy jelly as the test meal to control chewing other than vegetable consumption while participants were asked to chew shredded cabbage at a fixed pace. Thus, future research is warranted to examine the effects of vegetable consumption during thorough chewing followed by a solid-type mixed meal on incretin secretion and glucose metabolism.

## Conclusion

The present study demonstrates that consuming shredded cabbage while chewing and pureed cabbage without chewing appears to have no effect on postprandial glucose concentration; however, chewing on its own increases postprandial insulin, GIP and total GLP-1 concentrations in healthy young men. These findings lend support to the notion that consuming vegetable with chewing before a meal augments insulin secretion and extend the current literature that this augmentation of postprandial insulin release was concomitant with increased incretin hormones. Given that insulin secretion typically decreases with ageing, the finding of the study implies the importance of chewing even in healthy young adults.

## Data Availability

Some or all data sets generated during and/or analysed during the present study are not publicly available but are available from the corresponding author on reasonable request.
